# Reliability and Construct Validity of the Psychopathic Personality Inventory-Revised in a Swedish Non-Criminal Sample – A Multimethod Approach including Psychophysiological Correlates of Empathy for Pain

**DOI:** 10.1371/journal.pone.0156570

**Published:** 2016-06-14

**Authors:** Karolina Sörman, Gustav Nilsonne, Katarina Howner, Sandra Tamm, Shilan Caman, Hui-Xin Wang, Martin Ingvar, John F. Edens, Petter Gustavsson, Scott O Lilienfeld, Predrag Petrovic, Håkan Fischer, Marianne Kristiansson

**Affiliations:** 1 Department of Clinical Neuroscience, Karolinska Institutet, SE-171 77, Stockholm, Sweden; 2 Stress Research Institute, Stockholm University, SE-106 91, Stockholm, Sweden; 3 Aging Research Center, Karolinska Institutet and Stockholm University, SE-113 30, Stockholm, Sweden; 4 Department of Psychology, Texas A&M University, College Station, TX, 77843, United States of America; 5 Department of Psychology, Karolinska Institutet, SE-171 77, Stockholm, Sweden; 6 Department of Psychology, Emory University, Atlanta, GA, 30322, United States of America; 7 Department of Psychology, Stockholm University, SE-106 91, Stockholm, Sweden; University of Vienna, School of Psychology, AUSTRIA

## Abstract

Cross-cultural investigation of psychopathy measures is important for clarifying the nomological network surrounding the psychopathy construct. The Psychopathic Personality Inventory-Revised (PPI-R) is one of the most extensively researched self-report measures of psychopathic traits in adults. To date however, it has been examined primarily in North American criminal or student samples. To address this gap in the literature, we examined PPI-R’s reliability, construct validity and factor structure in non-criminal individuals (*N* = 227) in Sweden, using a multimethod approach including psychophysiological correlates of empathy for pain. PPI-R construct validity was investigated in subgroups of participants by exploring its degree of overlap with (*i*) the Psychopathy Checklist: Screening Version (PCL:SV), (*ii*) self-rated empathy and behavioral and physiological responses in an experiment on empathy for pain, and (*iii*) additional self-report measures of alexithymia and trait anxiety. The PPI-R total score was significantly associated with PCL:SV total and factor scores. The PPI-R Coldheartedness scale demonstrated significant negative associations with all empathy subscales and with rated unpleasantness and skin conductance responses in the empathy experiment. The PPI-R higher order Self-Centered Impulsivity and Fearless Dominance dimensions were associated with trait anxiety in opposite directions (positively and negatively, respectively). Overall, the results demonstrated solid reliability (test-retest and internal consistency) and promising but somewhat mixed construct validity for the Swedish translation of the PPI-R.

## Introduction

Psychopathy is associated with a constellation of affective, interpersonal, and behavioral traits including egocentricity, dishonesty, fearlessness, lack of empathy and guilt, and poor impulse control [1]. However, consensus is lacking about the role of certain core features of psychopathy. The nature and manifestation of antisocial behavior in relation to psychopathy remains unclear [1], however see [2] for findings that antisociality is an integral part of psychopathy across diverse international samples. Moreover opinions diverge regarding whether seemingly adaptive features (e.g., lack of anxiety, social poise, emotional resilience) are part of the psychopathy construct [1, 3, 4]. These conceptual uncertainties stem partly from the dominance of one measure in the field during the past three decades: the Psychopathy Checklist-Revised (PCL-R) [5–6]. The PCL-R is an extensively validated interview and file-based measure that captures both the affective/interpersonal traits and behavioral features of psychopathy. During the last decade, some critics have argued that the heavy focus on antisocial and criminal behavior in PCL-based instruments has contributed to an underemphasis on affective and interpersonal features in psychopathy (e.g., lack of empathy, fearlessness), unconfounded by criminality [7], but see [8] for a competing view.

To obtain an improved understanding of the conceptualization and correlates of psychopathic traits in different settings, several self-report measures for psychopathic traits in non-clinical samples have been developed, see [9] for a review. Moreover, new theoretical models of psychopathy have recently been developed that accord greater emphasis to affective and interpersonal psychopathic traits, with less emphasis on antisocial and criminal behavior [10–11]. To date, the Psychopathic Personality Inventory-Revised (PPI-R) [12] is the most widely researched self-report instrument for psychopathic traits in adults [13]. It has primarily been used in North American undergraduate or criminal samples [4], although some cross-cultural work is beginning to be conducted (e.g., [14–15]). Nevertheless, relatively little is known about the psychometric properties of this widely used measure in European nations.

In Sweden, previous research on psychopathy has mainly used PCL-based instruments to investigate violent behavior in institutionalized samples (e.g., [16–17]), or the Youth Psychopathic Traits Inventory (YPI) [18] to explore psychopathic traits in adolescent community samples (e.g. [19]). Research is needed on the prevalence and manifestation of psychopathic traits in non-criminal adults in Scandinavian samples. In this study, we translated the PPI-R into Swedish and investigated its psychometric properties; an important research endeavor given the dearth of research on cross-cultural manifestations of psychopathy (cf., [20–21]).

### The Psychopathic Personality Inventory-Revised (PPI-R)

The PPI-R is a well-validated measure designed to capture core affective and interpersonal dispositions of psychopathy (e.g., lack of empathy, guilt, and fear), with a less explicit focus on antisocial and otherwise deviant behaviors, which may be largely nonspecific to psychopathy [22]. The PPI-R is well-suited for use with both criminal and noncriminal groups [12]. It encompasses eight lower order factors/content scales [12] which some investigators have organized into two higher-order factors (e.g., [23]): 1) Fearless Dominance (FD), encompassing the subscales Social Influence (SOI), Fearlessness (F) and Stress Immunity (STI), 2) Self-Centered Impulsivity (SCI), encompassing the subscales Machiavellian Egocentricity (ME), Rebellious Nonconformity (RN), Blame Externalization (BE), and Carefree Nonplanfulness (CN). Several studies have failed to replicate this two-factor structure, however, [15], or have proposed alternative factor structures [24]. In the two-factor structure, the Coldheartedness subscale (CH) has not loaded highly on either factor and tends to be discarded from analyses. The CH scale is proposed to be part of a wider personality domain with several content areas of relevance to empathic ability (e.g., affective detachment, sentimentality and guilt) [25]. At present however, the precise conceptualization of the CH scale remains uncertain and research is needed on its correlates using assessments from several domains (e.g., personality based, behavioral, physiological) [25].

Across studies, the PPI-R higher order factors tend to be orthogonal or weakly correlated, and are often associated with measures of personality and behavior in opposite directions [23, 26]. For example, FD has been associated with positive social adjustment and an outgoing personality style (e.g., extraversion, venturesomeness) and low levels of anxiety, but also certain maladaptive outcomes including self-reported narcissism. In contrast, SCI has been associated with neuroticism and negative emotionality as well as externalizing behaviors (e.g., aggression and antisocial behavior) [23, 27].

In this study, the construct validity of the PPI-R was investigated in relation to self-report measures of theoretically relevant criterion variables: primarily empathy; but also alexithymia and anxiety-proneness in subgroups of participants. Associations between psychopathic traits and empathic ability were further investigated in relation to behavioral and physiological measures in an experiment examining empathy for pain [28]. The investigation of relations among measures drawn from different domains (e.g., self-report and interview based, behavioral and biological variables) is in line with a contemporary movement in broader personality research emphasizing the need to explore neurobiologically influenced transdiagnostic mechanisms [29–30]. Consistent with this approach, the “dual process model” proposes that the affective/interpersonal and impulsive/antisocial component of psychopathy are underpinned by separate etiological mechanisms (i.e., trait fearlessness and externalizing vulnerability, respectively) characterized by distinct physiological correlates [30–31]. Bolstering this theory, several studies investigating PPI-assessed psychopathic traits have demonstrated unique associations between elevated FD and diminished fear reactivity (cf. [14, 31–32]). Moreover, unique associations have been demonstrated between elevated SCI and different aspects of disinhibitory proneness, including higher skin conductance responses in a social stress test and diminished P3 amplitude [31, 33]. Nevertheless, the dual process model largely omits the role of emotional detachment, as assessed by the PPI CH scale. Given that emotional detachment, which includes impaired empathy, has long been proposed as one defining feature of psychopathy [34], research is needed on the psychophysiological correlates of the CH scale.

Current conceptualizations of empathy vary with respect to their emphasis on different components of the emotional response (e.g., affective response, emotional contagion, sympathy, cognitive perspective taking, motor empathy) [35]. More specifically, the nature of the emotional deficits in psychopathy is unclear. A prevailing view is that psychopaths demonstrate a specific deficit in emotional empathy [36]. Some recent findings, however, suggest that psychopathy may also be associated with an inadequate understanding of others’ beliefs and intentions, often referred to as cognitive empathy [37]. In this study, empathy for pain was examined in light of the underlying premise that a core neurophysiological network becomes activated not only while experiencing pain, but also when observing others in pain [38–39].

In addition to the associations with empathy, we investigated the construct validity of the PPI-R by examining its convergent and divergent associations with self-reported alexithymia and anxiety proneness. Alexithymia refers to an impaired ability to process, identify, and describe one’s emotions [40]. Overlap between the emotional and interpersonal deficits in alexithymia and certain features of psychopathy (e.g., lack of insight, warmth and empathy), has been proposed, although the nature of this association remains unclear [40]. The conceptual relevance of diminished anxiety proneness to the psychopathy construct is controversial [1, 13, 41–42]. Because the PCL-R was not explicitly designed to capture low anxiety as a feature of psychopathy, but see [43] for findings that low anxiety and fearlessness is indirectly incorporated in the PCL-R factors, the PPI-R and several other instruments which capture a broader range of emotional and social features of psychopathy, may be more relevant for investigating associations between psychopathic traits and anxiety [44]. Because the PPI (the predecessor of the PPI-R) was designed in part to capture some of the more presumably adaptive features of psychopathy [45], low anxiety-proneness is of theoretical relevance to the PPI-R nomological network. Previous research has demonstrated that SCI and FD are associated with self-rated anxiety in opposite directions (positively and negatively, respectively) [15].

## Aims

This study aimed to investigate reliability (test-retest and internal consistency), construct validity, and factor structure of the Swedish translation of the PPI-R. The PPI-R’s construct validity was investigated in subgroups of participants by examining its degree of overlap with (*i*) the Psychopathy Checklist: Screening Version (PCL:SV), (*ii*) self-rated empathy as well as behavioral and physiological responses in an experiment on empathy for pain, and (*iii*) additional self-report measures of alexithymia and trait anxiety. Based on previous literature [23], we hypothesized that scores on the PPI-R would be positively related to PCL:SV total scores, particularly with its affective/interpersonal Part 1. The CH-scale, which was developed to detect an absence of tender social emotions and a callous incapacity to sympathize with other’s suffering [12], was expected to demonstrate a negative association with self-rated emotional empathy and with behavioral/physiological outcomes in the empathy for pain experiment, but a positive association with alexithymia. Based on community research [15], we further hypothesized that SCI and FD would be associated with self-rated trait anxiety in opposite directions (positively and negatively, respectively).

## Materials and Methods

### Participants

The sample consisted of 227 individuals (81.1% men; *M* = 31.7 years, *SD* = 17.6, range 18–75). All participants were fluent in Swedish and reported no current or previous neurological/psychiatric illness or substance abuse. Most participants were pursuing university/college studies (*n* = 127, 57.2%), or had completed an advanced degree (*n* = 43, 19.4%) at the time of inclusion. Nine participants had missing information about educational background. Most participants (*n* = 184) were male. The nature and correlates of psychopathy in women remain controversial and largely unexplored [1, 46], but see [47–48], for findings that the manifestation and correlates of psychopathy may differ between genders.

Participants were recruited in four waves. Advertisements were posted at local campus areas and a freely accessible (i.e., not linked to a university) website on which studies seeking participants are listed (www.studentkaninen.se). In the first two waves, data were collected as part of a behavioral experiment in which the effects of benzodiazepines on empathy for pain were investigated. In Wave 1 (*n* = 85), participants were randomized to receive a one-time dose of 25 mg Oxazepam or placebo. In Wave 2 (*n* = 5), data were collected as part of a pilot experiment on the effect of a one-time dosage of 25 mg Midazolam. In Wave 3, participants were recruited by advertising on www.studentkaninen.se for a “study of personality”. In total, 62 potential participants received the PPI-R via mail, of whom 59 completed the form. In Wave 4, participants were recruited from a brain imaging study on the effects of sleep loss. 90 participants were invited to complete the PPI-R, and 83 did. The PPI-R was sent to participants by mail after their participation or handed over in person, and was completed by the participants in their own homes when they were not undergoing sleep deprivation. The Regional Ethical Review Board of Stockholm approved the studies (#2009/1128-31/3; 2009/1128-31/3; 2012/1098-31/2) and all participants provided informed consent.

Five participants had high scores (>45) on the Inconsistent Responding-40 (IR; described below) scale, which assesses random or careless responding (see “Measures”), and were therefore excluded [12]. Nineteen participants (8.4%) in the test- and two participants (3.8%) in the re-test session obtained an IR-40 score between 39 and 44, which could imply impaired validity of the PPI-R protocol [12].

### Measures

The *Psychopathic Personality Inventory-Revised* (PPI-R) [12] is a 154-item self-report measure using a Likert type scale (*1 = false*, *2 = mostly false*, *3 = mostly true*, *4 = true*). The PPI-R yields a total score as well as scores on eight content scales. The PPI-R also includes three validity scales; Deviant Responding, Virtuous Responding, and Inconsistent Responding-40, measuring aberrant responding/malingering, socially desirable responding, and random or careless responding, respectively. We translated the PPI-R and an authorized translator, blind to the original version, conducted back-translation. The back-translation was evaluated by the author of the original test and 10th author of this paper (SOL). The final translation was approved by the test publisher (Psychological Assessment Resources; PAR).

The *Interpersonal Reactivity Index* (IRI) [49] is a 28-item self-report measure divided into four scales developed to capture different aspects of empathy: Empathic Concern (EC), reflecting other-oriented feelings of warmth, compassion, and concern: Perspective Taking (PT), reflecting a tendency to understand and adopt other’s psychological view; Personal Distress (PD), reflecting self-oriented feelings of anxiety and discomfort in tense interpersonal situations; and Fantasy (FS), reflecting a tendency to imagine oneself in fictional situations. Factor analysis of the Swedish version showed that EC, PT and FS formed a common factor, whereas PD formed a largely separate factor [50].

The 20-item *Toronto Alexithymia Scale* (TAS-20) [51–52] encompasses three factors; Difficulty Identifying Feelings (DIF), reflecting difficulties distinguishing feelings from bodily sensations of emotions; Difficulty Describing Feelings (DDF), reflecting an inability to communicate feelings to other individuals; and Externally Oriented Thinking (EOT), reflecting a cognitive preoccupation with the details of external events, rather than content associated with feelings and fantasies. Each item is rated on a 5-point Likert scale (from *1 = strongly disagree* to *5 = strongly agree*). The scale has been validated in Swedish [53].

The *State-Trait Anxiety Inventory* (STAI) [54] is a widely used 40-item self-report instrument for measuring anxiety in adults. It consists of two scales: the State Anxiety Scale (STAI-S), measuring current anxiety by asking respondents to rate how they feel at present, and the Trait Anxiety Scale (STAI-T), which assesses anxiety-related manifestations over extended periods by asking people how they typically feel. Items are scored using a 4-point Likert Scale (from *0 = almost never* to *4 = almost always*).

### PCL:SV interviews

The *Psychopathy Checklist*: *Screening Version* (PCL:SV) [55] is a well-validated derivative measure of the PCL-R. It was developed for use with non-forensic samples including psychiatric and community groups [6]. The PCL:SV encompasses 12 items that are scored using a 3-point scale (*0 = clearly not present*, *1 = maybe present*, *2 = clearly present*). The items are parsed into two higher order factors: Part 1, reflecting affective and interpersonal features, and Part 2, reflecting social deviance.

A subgroup of randomly selected participants (*n* = 51) from waves 1–3 were invited to participate in a PCL:SV interview. Interviews were conducted by the first author (KS). For inter-rater reliability purposes, a random sub-sample of participants (*n* = 11) underwent a second, independent rating by another author (KH). Both interviewers underwent formal PCL-R training and were blind to all other self-reported data when they performed the interviews.

### Behavioral and physiological measures of empathy for pain

Participants in wave 1 underwent a behavioral experiment using a well-researched paradigm for empathy for pain [39, 56]. In this experiment, participants received pain stimulation by electrical shocks to the forearm alternately with a confederate seated next to them, and we recorded their rated unpleasantness, skin conductance responses (SCRs), heart rate (only in a subsample), and activity of the superciliary corrugator muscle using electromyography (EMG). Detailed methods and main results have been reported elsewhere [28]. Empathic responses refer here to responses to viewing painful stimuli to the other person, compared with receiving shocks oneself. For the purpose of the present study, we examined the associations between the PPI-R higher order factors, including the CH scale, on the one hand, and experimental outcomes, on the other. In total, 61 participants were included in the behavioral experiment. Reasons for exclusion were as follows: pilot participants (*n* = 8); failure to understand instructions, or reported suspicions at debriefing that the confederate was not a naïve participant, or failure to reach sufficiently high pain (VAS 80, *n* = 14), exclusion due to technical problems (*n* = 1), and markedly high score (>45) on the Inconsistent Responding-40 subscale (see above) (*n* = 1).

### Missing data

Scales on any of the self-report measures with more than 20% missing item responses (two IRI-protocols, i.e. 1.2%, and four TAS-20 protocols, i.e. 2.4%) were excluded. Scales with less than 20% missing items were prorated based on means of completed items in the respective subscales (21 PPI-R test protocols, i.e. 9.2%; six PPI-R re-test protocols, i.e. 12.5%; one IRI-protocol, i.e. 0.6%, and three TAS-20 protocols, i.e. 1.8%).

### Analyses

Construct validity was investigated by correlating PPI-R subscales to the other measures included in the study. Interrater reliability of PCL:SV ratings was determined using intraclass correlation coefficients (ICCs) [57]. We used a two-way random effects model (ICC_A1_) for a single rater with absolute agreement [58]. Independent samples *t*-tests were used to examine differences in PPI-R total scores between (a) participants who completed the PPI-R retest and those who did not and (b) participants who underwent a PCL:SV interview and those who did not. Cronbach’s α was calculated using R version 2.15.2 (2012) with the ltm package [59], with confidence intervals based on a bootstrap procedure with 1000 iterations.

Factor analyses were performed using Exploratory Structural Equation Modeling (ESEM) [60] to compare alternative factor structures of the PPI-R in Swedish. ESEM was performed using z-scores standardized for males and females separately. We tested four different models, based on previous factor analyses of the PPI [23, 24]. The first model (1a) had two factors encompassing FD (including the subscales SOI, F, STI) and SCI (including the subscales ME, RN, CN, BE), proposed by Benning and co-authors [23]. The second model (1b) permitted the subscale F to cross-load, given that it tends to load positively on both FD and SCI (e.g., [23]). The third model (2a) was a 3-factor model encompassing Factor 1 (SOI, STI); Factor 2 (ME, RN, BE, F); and Factor 3 (CH, CN) based on [24]. The fourth model (2a) was a restricted two-factor model, in which the CH scale was dropped and several alterations were made to the Factor-2 scales: RN and F were permitted to cross load on Factor 1, and STI had a negative loading on Factor 2, also proposed by [24]. Estimations were carried out in the Mplus program version 7.1 using the maximum likelihood estimator [61] with the target rotation [60]. Model fit was evaluated by the χ^2^, Comparative Fit Index (CFI), Root Mean Square Error of Approximation (RMSEA) and the Standardized Root Mean Square Residual (SRMR). CFI values > .95, RMSEA < .06, and SRMR < .08 are generally considered indicators of adequate fit [62]. These cut-offs were used as rule of thumb to compare the fit for the different models of PPI-R subscale associations.

Associations between the PPI-R higher-order factors and the behavioral/physiological measures were calculated using mixed-effects models, as there were repeated measures from each participant. Analyses were performed in R with the nlme package [63]. Standardized regression coefficients were estimated for the PPI-R factors and the CH scale, as predictors of empathic responding.

## Results

### PPI-R descriptive statistics

The average PPI-R total score was 291 (*SD* = 32.9, range 201–368). Males obtained higher total scores on the PPI-R (*M* = 297.10, *SD* = 31.83) than females (*M* = 266.0, *SD* = 24.95); *t*(225) = 5.99, *p*< .01, Cohen’s *d* = 1.09.

### Reliability: Test-retest and internal consistency

The PPI-R was mailed to all participants from Wave 1 for which current addresses were known (*n* = 82). Forty-eight individuals returned it (58.5%). Test-retest intervals averaged 95.5 days (range 6–199 days, *SD* = 46.3). Participants who did not complete the retest had higher PPI-R raw scores in the test session (*M* = 313.72, *SD* = 32.24) than participants who completed the retest (*M* = 299.00, *SD* = 27.03); *t*(78) = 2.21, *p* = .03, Cohen’s *d* = .49. PPI-R retest scores ranged from 240 to 377 (*M* = 300.31, *SD* = 31.05). Total score test-retest correlation was .89, *p*<.01. Test-retest correlations for PPI-R subscales ranged from .79 (CN) to .94 (F and FD), all *p* <.01. All scales demonstrated good internal consistency (α = .80-.87, see Table 1), although the value for CH was slightly lower (α = .77).

**Table 1:** Internal Consistency for the PPI-R Content Scales.

PPI-R scales	Cronbach’s α	95% CI
**Machiavellian Egocentricity**	.85	.81–.87
**Rebellious Nonconformity**	.83	.79–.85
**Blame Externalization**	.87	.84–.89
**Carefree Nonplanfulness**	.80	.76–.83
**Social Influence**	.86	.83–.88
**Fearlessness**	.82	.77–.85
**Stress Immunity**	.83	.79–.86
**Coldheartedness**	.77	.73–.81

*Note*.*n* = 227. PPI-R = Psychopathic Personality Inventory-Revised.

### PPI-R construct validity

#### Associations between PPI-R and PCL:SV

The PPI-R’s construct validity was investigated primarily by examining associations between PPI-R and PCL:SV with Pearson correlations. PCL:SV total scores ranged between 0–15 (*M* = 1.49, *SD* = 2.75); Part 1 ranged between 0–10 (*M* = .57, *SD* = 1.69) and Part 2 ranged between 0–7 (*M* = .92, *SD* = 1.53). Most participants (72.5%) obtained a total score of 0 or 1 and only six (11.8%) obtained a score of 5 or higher. No participant reached the suggested cutoff score for psychopathy (≥18). One participant obtained a score of 15 which is above the suggested cutoff for “possible psychopathy” [55]. Interrater reliability (ICC_A1_) was .90 for the total score, .92 for Part 1 and .85 for Part 2. PPI-R total scores at the test session did not differ significantly between participants who received a PCL:SV interview (*M* = 299.62, *SD* = 32.08) and participants who did not receive a PCL:SV interview (*M* = 300.83, *SD* = 31.12); *t*(144) = .22, *p* = .83, Cohen’s *d* = .04.

The two major PPI-R higher-order factors were significantly correlated, although this association was only small to medium in magnitude (*r* = .26, *p*<.01). The PPI-R total score demonstrated moderate correlations with PCL:SV total and Part 1 (Table 2). Neither FD nor CH demonstrated significant associations with PCL:SV total or factor scores. One outlier had a disproportionate impact on the PCL:SV total score as well as the score for Part 1 (e.g., the standardized residual for PPI-R total score and PCL:SV Part 1 was 5.11), and was therefore removed from the analysis.

**Table 2:** Pearson Correlations between PPI-R and PCL:SV, Total and Factor Scores (*n* = 50).

PPI-R	PCL:SV Total	Part 1	Part 2
**Total**	.38**	.43**	.21
**Fearless Dominance**	.13	.15	.05
**Self-Centered Impulsivity**	.43**	.48**	.27
**Coldheartedness**	.13	.19	.04

*Note*. Results presented without the outlier. PPI-R = Psychopathic Personality Inventory-Revised; PCL:SV = Psychopathy Checklist-ScreeningVersion.

* *p* < .05,

** *p* < .01.

#### Associations between PPI-R factors and criterion measures

The PPI-R CH-subscale demonstrated a strong negative association with the IRI-scale Empathic Concern (EC). It also demonstrated small to moderate negative associations with the three remaining empathy subscales (i.e., Perspective Taking, Personal Distress, Fantasy; Table 3). In the behavioral experiment, CH predicted low responses in empathy for pain on three out of four outcomes: rated unpleasantness, skin conductance responses, and corrugator activity (Table 4). Further regarding the empathy indices, FD was associated with the empathy subscales PT and PD in opposing directions (positively and negatively, respectively). Moreover, in the empathy experiment, FD predicted high self-rated unpleasantness in empathy for pain. SCI demonstrated a small positive association with the Fantasy subscale of the IRI, but small negative associations with the subscales Empathic Concern and Perspective Taking. Regarding the remaining criterion measures, the PPI-R factors SCI and FD were associated with trait anxiety in opposing directions (positively and negatively, respectively). Finally, FD demonstrated a moderate negative association with self-reported alexithymia (Table 3).

**Table 3:** Pearson Correlations between the PPI-R Factors, IRI-scales, STAI-T and TAS-20.

	IRI				STAI-T	TAS-20
	EC	PT	PD	FS		
**Fearless Dominance**	.05	.22**	-.41**	.14	-.21**	-.39**
**Self-Centered Impulsivity**	-.16*	-.21**	.15	.29**	.54**	-.10
**Coldheartedness**	-.63**	-.36**	-.26**	-.18*	.01	-.08

*Note*. IRI = Interpersonal Reactivity Index; EC = Empathic Concern; PT = Perspective Taking;

PD = Personal Distress; FS = Fantasy; STAI-T = State Trait Anxiety Inventory-Trait; TAS-20;

Toronto Alexithymia Scale. *n* varies between 159 and 163, due to some missing cases.

* *p* < .05,

** *p* < .01

**Table 4:** Associations between PPI-R subscales and behavioral/physiological responding in empathy for pain (standardized regression coefficient β [95% CI]), *n* = 61 for all outcomes except heart rate responses, for which *n* = 26.

	Rated unpleasantness	Skin conductance responses	Heart rate responses	Corrugator EMG responses
**Fearless Dominance**	.15 [.07, .23]***	-.01 [-.10, .08]	-.00 [-.20, .19]	.04 [-.04, .12]
**Self-Centered Impulsivity**	-.06 [-.15, .02]	.03 [-.06, .13]	-.06 [-.31, .19]	.05 [-.04, .14]
**Coldheartedness**	-.23 [-.31, -.15]***	-.14 [-.23, -.05]**	-.01 [-.21, .19]	-.08 [-.17, .00]

* *p* < .05,

** *p* < .01,

*** *p* < .001

#### PPI-R factor structure

Subscale correlations are shown in Table 5. With the 2-factor model as a reference [23], (a) subscales in the SCI-dimension (i.e, RN, BE, ME) demonstrated weak to moderate correlations, with *r*s = .13 –.55, (b) subscales in the FD-factor also correlated moderately (*r*s = .29-.35), and c) the F subscale correlated weakly to moderately with two of the SCI-scales (ME and RN). We analyzed the factor structure using four different ESEM models based on prior work. Two of them converged, but with poor fit (Table 6). We re-ran the ESEM-analyses with men separately. In line with the original finding, the results demonstrate that two of the models (1b and 2a) did not converge. The remaining models had a poor fit, with only minor changes in the fit indices (Table A in S1 File).

**Table 5:** PPI-R subscale correlations total sample (*n* = 227).

Subscale	1	2	3	4	5	6	7	8
	SCI				FD			CH
**1.Rebellious Nonconformity (RN)**	-	.42**	.50**	.30**	-.16*	.29**	.46**	-.04
**2.Blame externalization (BE)**		-	.55**	.15*	-.20**	.19**	.13*	.05
**3.Machiavellian Egocentricity (ME)**			-	.13*	-.13	.34**	.34**	.25**
**4.Carefree Nonplanfulness (CN)**				-	-.09	-.15*	.07	.04
**5.Stress Immunity (STI)**					-	.30**	.29**	.28**
**6.Social Influence (SOI)**						-	.35**	.01
**7.Fearlessness (F)**							-	.18**
**8.Coldheartedness (CH)**								-

*Note*.

* *p* < .05,

** *p* < .01

**Table 6:** Fit indices for proposed PPI-R factor structures analysed by Exploratory Structural Equation Modeling (ESEM).

Model	Chi_2_	df	P	RMSEA	CFI	SRMR
**1a**	132.7	13	< .001	.201	.642	.116
1b*	-	-	-	-	-	-
2a*	-	-	-	-	-	-
**2b**	57.2	10	< .001	.144	.859	.061

*Note*.

*Model did not converge

## Discussion

This study made use of a multi-method approach that included (a) self-report measures (i.e., one measure of psychopathic traits and measures of criterion variables), (b) an interview based measure of psychopathic traits, and (c) behavioral and physiological indicators in an experiment of empathy for pain, to investigate the construct validity of the Swedish translation of the PPI-R. Previous research has examined cross-cultural manifestations of psychopathic traits in criminal and non-criminal samples using the PCL-R and its derivatives, including self-report measures [64–66]. A relatively recent study examined self-reported psychopathic traits in a large sample (*N* = 33,016; mainly encompassing college students or individuals from the community) from multiple world regions [66]. In this study, psychopathic traits were assessed with the Self-Report Psychopathy (SRP) [67] scale, and the results demonstrated cultural differences in the expression of psychopathic traits. For example, individuals with elevated levels on the affective SRP-facet were more common among females from Western Europe, compared to females from other world regions [66]. Cross-cultural research on the PPI-R is lacking to date however. More specifically, research is needed in Scandinavian non-criminal adult samples. The findings in the current study contribute to knowledge regarding the PPI-R’s psychometric properties in a Swedish context, in which it has not been previously examined in research. Our mean PPI-R scores were comparable to those of a previous community study from Belgium [15] (Table B in S1 File). They are also lower than what has been reported from several delinquent samples (cf. [24, 68]). Our findings also add to knowledge regarding the correlates of psychopathic traits that are relevant to broader conceptualizations of psychopathy. This goal is important given that the PPI-R encompasses features (e.g., fearlessness, lack of anxiety) that have been emphasized in historical definitions of psychopathy but that are not explicitly included in PCL-based measures.

Overall, the results support reliability and construct validity of the PPI-R in a Swedish non-criminal setting. Reliability (test-retest and internal consistency) scores were similar to those obtained in the original validation studies [12]. The PPI-R’s construct validity was largely supported by its positive correlation with the PCL:SV total and factor scores, which corroborates our hypothesis and replicates previous research [44]. The study also contributes new data regarding the correlations of the PPI-R CH-scale, a measure that remains underresearched and poorly understood [25].

### Construct validity: associations with the PCL:SV

To evaluate construct validity, it is important to investigate convergent and discriminant associations between the target construct and theoretically relevant measures [68]. In our sample, SCI was the only PPI higher-order factor that correlated significantly with the PCL:SV and its factors. This finding dovetails with research suggesting that externalizing factors account largely for the covariation between PPI-R and PCL-R/SV [68]. SCI also demonstrated a correlation with PCL:SV Part 1 in the moderate to large effect range (*r* = .43). This finding ran counter to our prediction, as well as recent meta-analytic work demonstrating only modest correlations (average *r* = .21) between SCI and PCL-R F1 [26].

To explore this unexpected finding, we conducted supplementary analyses which revealed that the PPI-R subscale Machiavellian Egocentricity (ME) accounted for most of this covariance. This finding may be interpreted within the framework of the recently formulated triarchic model of psychopathy [11]. According to this model, ruthless exploitation of others, captured in the ME subscale, is encompassed within the triarchic domain of meanness (reflecting tendencies of callousness, lack of empathy, and aggression) and also in PCL-R factor 1 [11]. However, CH, which is proposed to reflect aspects of meanness not captured by SCI [11], did not demonstrate a significant correlation to the PCL:SV or its factor scores. This is an unexpected finding given that meta-analytic work has demonstrated at least modest positive associations between CH and both PCL-R factors [26]. More specifically, a preferential correlation has been demonstrated between CH and affective domains of psychopathy measures, assessing deficient empathy and guilt [15, 44]. Another notable finding is that FD did not correlate significantly with the PCL:SV total or its factor scores. We predicted a preferential association between FD and PCL:SV Part 1, given that they both ostensibly assess core traits within Cleckley’s [69] conceptualization of psychopathy. Previous research has demonstrated at least modest associations between FD and PCL-R Factor 1, ranging from .20 and .45 [3]. Even though the lack of convergence in our study is surprising it is important to keep in mind that the different instruments are based on partly diverging conceptualizations of psychopathy. More specifically, the affective/interpersonal features in PCL-based assessments reflect largely maladaptive features of psychopathy. In contrast, the subscales in the FD factor encompass traits (fearlessness, stress immunity, social poise) that are more explicitly adaptive. These quasi-adaptive features are also reflected in the boldness domain of the triarchic model of psychopathy [11]. Even though the construct validity of PPI-FD/boldness remains controversial, given that it appears to assess aspects of positive adjustment not captured by other measures [4, 26], conceptualizations of PPI-FD/boldness are based on seminal models of psychopathy that emphasize certain adaptive characteristics, such as lack of nervousness and superficial charm (e.g., [69]). The relevance of boldness to the psychopathy construct was supported by a recent meta-analysis of 32 samples. The review yielded medium to large correlations between measures of boldness and non-PCL based psychopathy measures, which in contrast to the PCL, were not developed and validated in prison samples, which tend to be characterized by largely unsuccessful individuals [70]. In light of these partly divergent conceptualizations o psychopathy, the lack of overlap between PPI-R and PCL:SV should be interpreted in light of the degree to which the latter is used as a “benchmark” for psychopathy [44, 71].

### Additional self-rated criterion measures: empathy and anxiety

In line with our hypothesis, CH demonstrated significant negative associations with all four empathy subscales. This finding suggests that CH may assess deficient reactivity to others’ emotional states in general. Furthermore in line with our expectations, SCI and FD were significantly associated with trait anxiety in opposite directions (positively and negatively, respectively). This finding replicates previous research [26] and suggests that trait anxiety is relevant to some features of the psychopathy construct. Even though a conceptual debate is ongoing regarding the role of deficient anxiety to psychopathy [1, 41–43], lack of anxiety has been emphasized in historical definitions of psychopathy (cf. [72]), and is explicitly covered in the emergent conceptual models of psychopathy, particularly in the boldness domain in the triarchic model of psychopathy [11]. Moreover, recent research on psychopathic traits in community groups has indicated that trait anxiety could be a marker to distinguish between different subgroups of individuals with elevated levels of psychopathic traits [73]. Therefore, more knowledge is needed on the association between trait anxiety and psychopathic traits in different groups.

FD correlated positively with one empathy scale (PT), and negatively to PD. Previous research has demonstrated a negative association between FD and emotional empathy [74], but see [15] for a partial positive correlation between FD and empathy total score encompassing both cognitive and emotional reactivity. In addition, FD was negatively associated with alexithymia, which may reflect the tendency of individuals with alexithymia to be prone to anxiety [40].

### Behavioral and physiological criterion measures: empathy for pain

Perceiving pain in others is considered to be a necessary component of empathic concern and a motivating factor for prosocial behavior [75]. Brain imaging research has demonstrated altered activity in neural networks crucial to empathic processing in psychopathic offenders compared to controls [75]. Skin conductance responses (SCRs) is an established physiological indicator of the affective component of empathy [76]. Attenuated emotional processing assessed with SCRs has been demonstrated in offenders with various psychiatric diagnoses however without psychopathy [77]. In a similar vein, a recent study on empathy for pain demonstrated reduced SCRs following the experimental stimuli in incarcerated offenders compared to controls, regardless of the offenders’ degree of psychopathic traits [76]. In this same study, degree of PPI-assessed psychopathic traits in the control subjects were unrelated to SCRs [76]. It is important to further investigate emotion processing in relation to different subfactors of psychopathy, given that they might be underpinned by different etiological processes [78]. Research is lacking on physiological correlates of the CH-scale specifically. In our study, the CH-scale predicted lower rated unpleasantness and smaller SCRs to others’ pain, further supporting the validity of the subscale. This is in line with previous research demonstrating a negative association between elevated scores on the CH-scale and motor cortex excitability in an experiment on empathy for pain among healthy community participants [79].

### Factor analyses

In this study, we used the relatively novel technique of ESEM. ESEM, which is a combination of confirmatory and exploratory factor analysis, allows for improved modeling flexibility [60]. Although we investigated several previously proposed factor structure of the PPI-R [23–24], no structure was confirmed using traditional criteria for model fit. This finding is not entirely surprising [71] given that the PPI-R was not designed with a higher-order structure in mind [12]. Moreover, confirmatory factor analyses on the original English version have provided somewhat mixed findings (cf. [24]). Nevertheless, the PPI-R is hardly alone in this regard. Recent research has demonstrated that a number of widely used personality trait inventories tend to exhibit poor fit using stringent CFA criteria, partly due to cross-loadings of certain subscales [80]. With respect to the present findings, the Fearlessness subscale demonstrated correlations with two SCI-subscales: RN and ME. Cross loadings of the Fearlessness scale has been demonstrated in previous research [24, 74, 81]. This finding is interesting in light of the ongoing debate regarding whether FD reflects aspects of positive adjustment (cf. [82]), or whether it also encompasses maladaptive features captured by SCI.

### Limitations

Despite its strengths, including our use of multiple methods, this study was marked by several limitations. In particular, the results are not easily generalizable to broader community groups, due to highly selected participants (i.e., mainly degree holders). Another potential limitation of the study is its inclusion of a mixed gender sample. In principle, some of our positive findings could have been spurious, reflecting an “admixture problem”, in which a correlation between two variables emerges when combining two samples in which these variables are uncorrelated [83]. To address this concern, in subsidiary analyses we conducted subgroup analyses by gender (Tables C-D in S1 File) and found that there are instances (e.g., the correlation between FD and IRI-EC), where the results for the entire group is inflated or deflated. Due to these additional tests, we report both uncorrected significance and false discovery rate (FDR)-corrected significance (Tables C-D in S1 File). Overall however, the subgroup analyses did not change substantively the interpretations of the correlations. It is also worth noting that several previous studies on PPI-R assessed subclinical psychopathic traits have used mixed-gender samples (e.g., [84–85]). Hence, our major positive results appear to apply to both men and women. To account for the multitude of tests, the analyses in Tables 2 and 4 were also re-computed using FDR-corrected significance (Tables E-F in S1 File).

Some of the analyses were underpowered. Overall therefore, results that replicate or contradict previous research might be due to the relatively small sample size. Findings regarding the PPI-higher order factors should be interpreted with some caution, given that the factor analysis failed to demonstrate the two-factor structure previously proposed [23]. The lack of factor structure replication could reflect problematic aspects on the item level, given that researchers thus far have examined the PPI-scales in factor analyses [24, 86]. Our sample was not large enough, however, to investigate factor loadings of individual items. Future studies should investigate the Swedish translation of the PPI-R in large community and correctional samples, including both men and women.

Future studies would benefit from additional behavioral correlates of the criterion variables of interest, such as indices of physical aggression.

## Conclusion

This study demonstrated good reliability and promising but somewhat mixed construct validity for the Swedish translation of the PPI-R. Even though the factor findings should be interpreted with some caution given the failure to replicate the proposed factor structure, the results provide preliminary evidence that the PPI-R higher-order factors reflect distinct dimensions given their convergent and discriminant relations with external variables of relevance to psychopathy.

## Supporting Information

S1 File TABLE A, Fit indices for proposed PPI-R factor structures analysed by Exploratory Structural Equation Modeling (ESEM), in a separate analysis on males. TABLE B, PPI and PPI-R comparison figures from previous studies. TABLE C, Pearson correlations between the PPI-R Factors, IRI-scales, STAI-T and TAS-20 values reported for males and females separately. TABLE D, PPI-R subscale correlations total sample, with values reported for males (n = 184) and females (n = 43) separately. TABLE E, Pearson Correlations between PPI-R and PCL:SV, Total and Factor Scores (*n* = 50). TABLE F, Associations between PPI-R subscales and behavioral/physiological responding in empathy for pain (standardized regression coefficient *β* [95% CI]), *n* = 61 for all outcomes except heart rate responses, for which *n* = 26.
(DOC)
