# Supplementary material for: Reliability and Construct Validity of the Psychopathic Personality Inventory-Revised in a Swedish Non-Criminal Sample – A Multimethod Approach including Psychophysiological Correlates of Empathy for Pain
Source: PLoS One. 2016 Jun 14;11(6):e0156570. doi: 10.1371/journal.pone.0156570 (PMC4907435; doi:10.1371/journal.pone.0156570)
Supplement: S1 File — TABLE A, Fit indices for proposed PPI-R factor structures analysed by Exploratory Structural Equation Modeling (ESEM), in a separate analysis on males. TABLE B, PPI and PPI-R comparison figures from previous studies. TABLE C, Pearson correlations between the PPI-R Factors, IRI-scales, STAI-T and TAS-20 values reported for males and females separately. TABLE D, PPI-R subscale correlations total sample, with values reported for males (n = 184) and females (n = 43) separately. TABLE E, Pearson Correlations between PPI-R and PCL:SV, Total and Factor Scores (n = 50). TABLE F, Associations between PPI-R subscales and behavioral/physiological responding in empathy for pain (standardized regression coefficient β [95% CI]), n = 61 for all outcomes except heart rate responses, for which n = 26. (DOC) [file pone.0156570.s001.doc]

**TABLE A. Fit indices for proposed PPI-R factor structures analysed by Exploratory Structural Equation Modeling (ESEM), in a separate analysis on males.**

| **Model** | **Chi_2_** | **df** | **P** | **RMSEA** | **CFI** | **SRMR** |
| --- | --- | --- | --- | --- | --- | --- |
| **1a** | 101.0 | 13 | <.001 | .192 | .672 | .110 |
| **1b*** | - | - | - | - | - | - |
| **2a*** | - | - | - | - | - | - |
| **2b** | 49.98 | 10 | <.001 | .147 | .851 | .062 |

*Note*. *Model did not converge

**TABLE B. PPI and PPI-R comparison figures from previous studies**

| **Sample** | **PPI-R total score** | | **Fearless Dominance** | | **Self-Centered Impulsivity** | | **Coldheartedness** | |
| --- | --- | --- | --- | --- | --- | --- | --- | --- |
|  | **M** | **SD** | **M** | **SD** | **M** | **SD** | **M** | **SD** |
| **Community samples^a^** |  |  |  |  |  |  |  |  |
| Uzieblo et al., 2010. Mixed-gender sample, *N* = 675 | 276.96 | 34.38 | 109.17 | 19.84 | 135.16 | 21.01 | 32.62 | 6.58 |
| **Delinquent samples^b^** |  |  |  |  |  |  |  |  |
| Malterer et al., 2010  (a) Incarcerated males (*N* = 876)* | 380.24 | 41.61 |  |  |  |  | 47.02 | 9.44 |
| Malterer et al., 2010  (a) Incarcerated males (*N* = 247)* | 386.74 | 40.33 |  |  |  |  | 48.01 | 9.96 |
| Neumann et al., 2008  Incarcerated males (*N* = 1224)* |  |  |  |  |  |  | 47.19 | 9.56 |
|  |  |  |  |  |  |  |  |  |

^a^The total PPI-R scores for the normative community/college samples reported in the test manual are as follows: 301.06 (M), 31.26 (SD) for males aged 18-24 years (*n* = 235); 300.65 (M), 32.83 (SD) for males aged 25-29 years (*n* = 34); 289.66 (M), 34.23 (SD) for males aged 30-39 years (*n* = 56), and 284.29 (M), 33.07 (SD) for males aged 40-49 years (*n* = 34). ^b^The total score for the PPI-R normative offender sample (*N* = 154) was 283.86, 28.99 (SD).

* PPI

**TABLE C. Pearson correlations between the PPI-R Factors, IRI-scales, STAI-T and TAS-20 values reported for males and females separately.**

|  | **IRI** | | | | **STAI-T** | **TAS-20** |
| --- | --- | --- | --- | --- | --- | --- |
|  | **EC** | **PT** | **PD** | **FS** |  |  |
| **Fearless Dominance** | .05  M =.21*  F= .24 | .22**/*  M = .34**  F = .16 | -.41**  M = -.40**  F = -.36*/ns | .14  M = .23*  F = -.05 | -.21**/*  M = -.33**  F = -.16 | -.39**  M = -.36**  F = -.15 |
| **Self-Centered Impulsivity** | -.16*/ns  M = -.04  F = -.09 | -.21**/*  M = -.20*/ns  F = -.10 | .15  M = .18  F = .34*/ns | .29**  M = .36**  F = .18 | .54**  M = .52**  F = .51**/* | -.10  M = -.10  F = .43**/* |
| **Coldheartedness** | -.63**  M = -.60**  F = -.43**/* | -.36**  M = -.36**  F = -.23 | -.26**  M = -.16  F = -.37*/ns | -.18*  M = -.12  F = -.34*/ns | .01  M = .01  F = -.34*/ns | -.08  M = .12  F= -.31*/ns |

*Note.* IRI = Interpersonal Reactivity Index; EC = Empathic Concern; PT = Perspective Taking;

PD = Personal Distress; FS = Fantasy; STAI-T = State Trait Anxiety Inventory-Trait; TAS-20;

Toronto Alexithymia Scale. *n* varies between 159 and 163, due to some missing cases.

* *p* < .05, ** *p* < .01

n males range between 116-120, n females = 43.

False discovery rate (FDR)-corrected significance: Where the significance level changed due to false discovery rate correction, the corrected significance level is shown after /.

**TABLE D. PPI-R subscale correlations total sample, with values reported for males (n = 184) and females (n = 43) separately**

| **Subscale** | **1** | **2** | **3** | **4** | **5** | **6** | **7** | **8** |
| --- | --- | --- | --- | --- | --- | --- | --- | --- |
|  | **SCI** | | | | **FD** | | | **CH** |
| **1.Rebellious Nonconformity (RN)** | - | .42**  M =.40**  F = .44** | .50**  M = .46**  F = .65** | .30**  M = .28**  F = .36*/ns | -.16*  M = -14  F = -.37*/ns | .29**  M = .30**  F = .13 | .46**  M = .42**  F = .49** | -.04  M = -.11  F = -.03 |
| **2.Blame externalization (BE)** |  | - | .55**  M = .52**  F = .43** | .15*  M = .17*  F = -.01 | -.20**  M = -.22**  F = -.41**/* | .19**  M = .15*/ns  F = .30 | .13*/ns  M = .04  F = .22 | .05  M = -.02  F = -.13 |
| **3.Machiavellian Egocentricity (ME)** |  |  | - | .13*/ns  M = .14  F = .11 | -.13  M = -.11*/ns  F = -.46**/* | .34**  M = .32**  F = .25 | .34**  M = .27**  F = .31*/ns | .25**  M = .19*  F = .06 |
| **4.Carefree Nonplanfulness (CN)** |  |  |  | - | -.09  M = -.09  F = -.12 | -.15*  M = -.14  F = -.27 | .07  M = .06  F = .07 | .04  M = .06  F = -.13 |
| **5.Stress Immunity (STI)** |  |  |  |  | - | .30**  M = .31**  F = .22 | .29**  M = .34**  F = .01 | .28**  M = .24**  F = .38* |
| **6.Social Influence (SOI)** |  |  |  |  |  | - | .35**  M = .37**  F = .12 | -.01  M = -.08  F = .07 |
| **7.Fearlessness (F)** |  |  |  |  |  |  | - | .18**  M = .13  F = -.06 |
| **8.Coldheartedness (CH)** |  |  |  |  |  |  |  | - |

*Note*. * *p* < .05, ** *p* < .01. False discovery rate (FDR)-corrected significance: Where the significance level changed due to false discovery rate correction, the corrected significance level is shown after /.

**TABLE E. Pearson Correlations between PPI-R and PCL:SV, Total and Factor Scores (*n* = 50)**

| **PPI-R** | **PCL:SV Total** | **Part 1** | **Part 2** |
| --- | --- | --- | --- |
| **Total** | .38**/* | .43** | .21 |
| **Fearless Dominance** | .13 | .15 | .05 |
| **Self-Centered Impulsivity** | .43** | .48** | .27 |
| **Coldheartedness** | .13 | .19 | .04 |

*Note.* False discovery rate (FDR)-corrected significance: Where the significance level changed due to false discovery rate correction, the corrected significance level is shown after /.

**TABLE F. Associations between PPI-R subscales and behavioral/physiological responding in empathy for pain (standardized regression coefficient *β* [95% CI]), *n* = 61 for all outcomes except heart rate responses, for which *n* = 26.**

|  | **Rated unpleasantness** | **Skin conductance responses** | **Heart rate responses** | **Corrugator EMG responses** |
| --- | --- | --- | --- | --- |
| **Fearless**  **Dominance** | .15 [.07, .23]*** | -.01 [-.10, .08] | -.00 [-.20, .19] | .04 [-.04, .12] |
| **Self-Centered**  **Impulsivity** | -.06 [-.15, .02] | .03 [-.06, .13] | -.06 [-.31, .19] | .05 [-.04, .14] |
| **Coldheartedness** | -.23 [-.31, -.15]*** | -.14 [-.23, -.05]**/* | -.01 [-.21, .19] | -.08 [-.17, .00] |

*Note. p* < .05, ** *p* < .01, *** *p* < .001 FDR: Where the significance level changed due to false discovery rate correction, the corrected significance level is shown after /.
